# Suramin Targets the Conserved Ligand-Binding Pocket of Human Raf1 Kinase Inhibitory Protein

**DOI:** 10.3390/molecules26041151

**Published:** 2021-02-21

**Authors:** Chenyun Guo, Zhihua Wu, Weiliang Lin, Hao Xu, Ting Chang, Yazhuang Dai, Donghai Lin

**Affiliations:** 1 Key Laboratory of Chemical Biology of Fujian Province, Department of Chemical Biology, College of Chemistry and Chemical Engineering, Xiamen University, Xiamen 361005, China; guochy78@xmu.edu.cn (C.G.); wuzh@xmu.edu.cn (Z.W.); wllin@stu.xmu.edu.cn (W.L.); xuhao1020@stu.xmu.edu.cn (H.X.); ctingmelody@163.com (T.C.); 2 Department of Medicinal Chemistry, China Pharmaceutical University, Nanjing 210009, China; daiyazhuang@163.com

**Keywords:** suramin, hRKIP, BLI, NMR, ERK phosphorylation

## Abstract

Suramin was initially used to treat African sleeping sickness and has been clinically tested to treat human cancers and HIV infection in the recent years. However, the therapeutic index is low with numerous clinical side-effects, attributed to its diverse interactions with multiple biological macromolecules. Here, we report a novel binding target of suramin, human Raf1 kinase inhibitory protein (hRKIP), which is an important regulatory protein involved in the Ras/Raf1/MEK/ERK (MAPK) signal pathway. Biolayer interference technology showed that suramin had an intermediate affinity for binding hRKIP with a dissociation constant of 23.8 µM. Both nuclear magnetic resonance technology and molecular docking analysis revealed that suramin bound to the conserved ligand-binding pocket of hRKIP, and that residues K113, W173, and Y181 play crucial roles in hRKIP binding suramin. Furthermore, suramin treatment at 160 µM could profoundly increase the ERK phosphorylation level by around 3 times. Our results indicate that suramin binds to hRKIP and prevents hRKIP from binding with hRaf1, thus promoting the MAPK pathway. This work is beneficial to both mechanistically understanding the side-effects of suramin and efficiently improving the clinical applications of suramin.

## 1. Introduction

Suramin, a polysulphonated napthylurea, was originally developed as a drug for clinically treating African trypanosomas and onchocerchiasis [1]. Owing to its capacity of inhibiting the reverse transcriptase activity of viruses, suramin was also clinically tested to treat HIV infection [2,3]. Additionally, suramin has been reported to have antitumor activity and can potentially be used in cancer therapy, especially in the treatment of prostate cancer [4,5,6,7]. However, the therapeutic index of suramin is low with numerous clinical side-effects. Previous works have demonstrated that suramin has growth-stimulatory effects on several cancer cell lines, including prostate cancer, lung cancer, and breast cancer [8,9,10,11,12]. The diverse and seemingly contradictory roles of suramin may be attributed to its multiple interactions with many biological macromolecules in vivo [13,14,15,16,17,18,19]. Although many clinical trials with suramin have been performed, the polypharmacology of suramin at the molecular level remains unclear [20]. Suramin analogues have been designed based on its interaction with targets to improve its activity, such as NF023, NF110, NF449, and so on [21,22]. Expectedly, a detail elucidation of intermolecular interactions between suramin and its potential targets would be beneficial to both mechanistically understanding the clinical side-effects of suramin and efficiently eliminating the side-effects by improving suramin and designing new analogues.

In our previous work, we determined the solution structure of human Raf1 kinase inhibitory protein (hRKIP) [23]. Based on the structural characteristics of hRKIP ligand-binding pocket, we screened out 44 potential ligands of hRKIP from a drug library by molecular docking software MOE. Within these ligands, suramin had the highest score of −11.57. The bioinformatic analysis suggested that suramin could potentially bind to hRKIP. As is known, hRKIP is the negative modulator of the MAPK (Ras/Raf1/MEK/ERK) signal pathway which plays crucial roles in cellular biological processes [24]. This pathway is activated by extracellular signal molecules, such as epidermal growth factor (EGF). The activated Ras kinase phosphorylates the Raf1 kinase, and the phosphorylated Raf1 kinase subsequently phosphorylates and activates the following MEK and ERK kinases [25]. The activation of ERK induces the expression of many downstream genes involved in the regulation of cell proliferation. The “open” and “close” statuses of the MAPK pathway are finely regulated in vivo and its dysregulation will cause many diseases [26]. It has been demonstrated that hRKIP can bind with human Raf1 kinase (hRaf1) through its conserved ligand-binding pocket and block the signal transduction [24]. Expectedly, the elimination of the hRKIP-hRaf1 interaction will disturb the regulatory role of hRKIP in the MAPK pathway, thereby leading to tumor occurrence, proliferation, differentiation, invasion, and metastasis [27,28,29,30,31,32,33].

In the present work, we found that suramin targeted hRKIP through binding to its conserved ligand-binding pocket, and significantly relieved the inhibitory effect of hRKIP on the MAPK signal pathway. Our results not only shed light on the molecular mechanisms of the side-effects of suramin, but also provide valuable information for both improving clinical applications of suramin and developing more efficient and specific suramin analogues.

## 2. Results and Discussion

### 2.1. Suramin has a Micromolar Affinity for hRKIP

**Figure 1:**
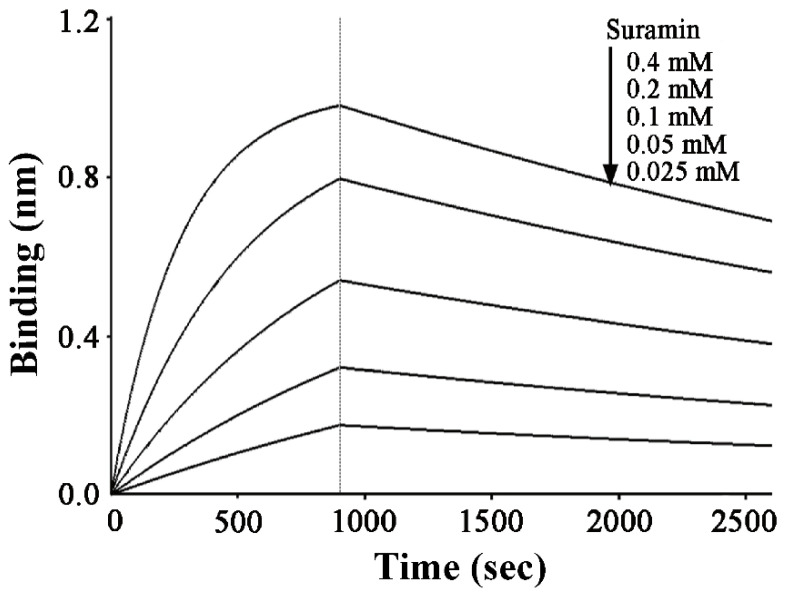
BLI assays of suramin binding to hRKIP.

We performed BLI experiments to detect the interaction of hRKIP with suramin, and quantitatively compared the affinities of hRKIP for binding suramin and its native substrate PE (*oO*-phosphorylethanolamine). Suramin exhibited concentration-dependent sensorgrams within the concentration range tested (Figure 1). The dissociation constant for hRKIP interacting with ligand was calculated by fitting the sensorgrams to a single-site binding model. Both the association rate (k_on_) and dissociation rate (k_off_) of PE interacting with hRKIP were measured to be 3.0 M^−1^ s^−1^ and 0.0264 s^−1^, respectively. Meanwhile, the k_on_ and k_off_ values for suramin with hRKIP were 8.69 M^−1^ s^−1^ and 0.0002 s^−1^, respectively, and the resulting dissociation constant KD of 23.8 µM was much smaller than KD value of 8.81 mM for PE binding to hRKIP [34]. This result indicated that suramin possessed an about 370-fold higher affinity for binding to hRKIP compared with PE acting as the native substrate of hRKIP.

### 2.2. Identification of Suramin Binding Sites on hRKIP

**Figure 2:**
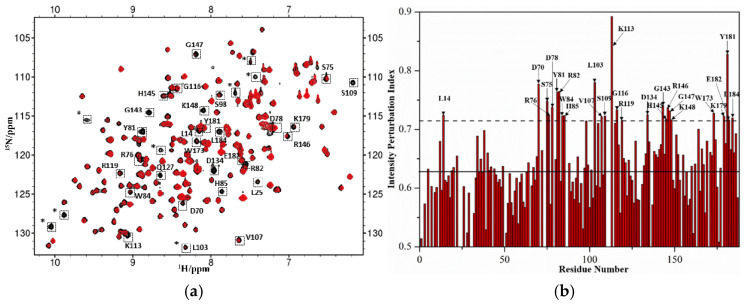

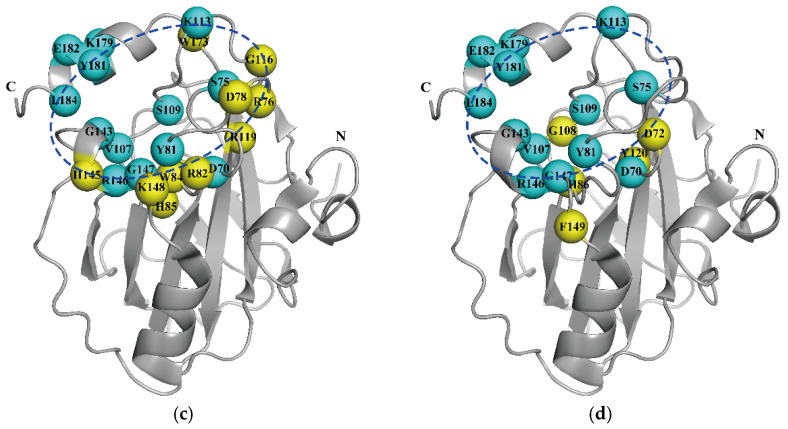
Binding sites of suramin on hRKIP determined by nuclear magnetic resonance (NMR) spectroscopy (**a**) Overlaid ^1^H-^15^N HSQC spectra of hRKIP in the absence (black) and presence (red) of suramin at 1:1 molar ratio. * represents unassigned peaks from the residues located at the linker between target gene and the vector. (**b**) The intensity perturbation indexes versus residue number. Solid line: the average value of intensity perturbation index; Dashed line: average + SD. (**c**) Residues with significant broadened peaks are mapped to the solution structure of hRKIP (PDB: 2L7W). Dashed circle: the ligand-binding pocket of hRKIP. (**d**) Residues involved in the hRKIP- hRaf1^NTD^ interaction are mapped to the same 3D structure [36]. Cyan balls: common residues involved in the interactions of hRKIP with both suramin and hRaf1^NTD^. Yellow balls: specific residues for the interaction of hRKIP with either suramin or hRaf1^NTD^.

2D ^1^H-^15^N HSQC spectrum recorded on ^15^N-labeled protein can provide information regarding the chemical shift and peak width for each non-proline residue, which are very sensitive to the chemical environment around the residue. Once the protein interacts with a ligand, residues involved in the intermolecular interaction will potentially experience changes in chemical shifts and/or peak widths, which indicate potential binding sites of the ligand on the protein. After suramin stock solution was added into the ^15^N-labeled hRKIP sample, we observed some peaks in the ^1^H-^15^N HSQC spectrum were significantly broadened due to suramin binding to hRKIP. These broadened peaks were identified to be residues L14, D70, S75, R76, D78, Y81, R82, W84, H85, L103, V107, S109, K113,G116, R119, D134, G143, H145, R146, G147, K148, W173, K179, Y181, E182, and L184 based on the available backbone assignments of hRKIP (Figure 2a,b) [35]. Except for L14 which is located at the *N*-terminal random coil, other significantly broadened peaks were associated with residues located in both the conserved ligand-binding pocket of hRKIP and the loop covering residues 127–149, indicating that suramin interacted with hRKIP primarily through binding to its conserved ligand-binding pocket at an intermediate affinity (Figure 2b,c). Notably, most of the residues associated with broadened peaks were consistent with the binding sites of hRaf1NTD on hRKIP, including D70, S75, Y81, V107, S109, K113, R146, K179, Y181, and E182 (Figure 2c,d) [36]. These results suggest that suramin and hRaf1NTD share basically similar binding sites on hRKIP.

### 2.3. Structural Model of Suramin Binding to hRKIP

**Figure 3:**
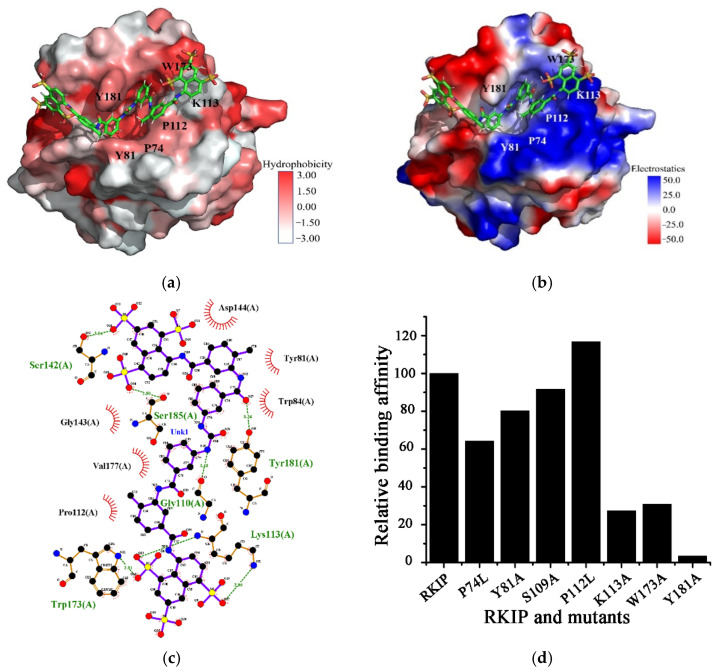
The key residues for hRKIP binding suramin. (**a**,**b**) Structural model of the hRKIP-suramin complex established by using the molecular docking approach based on the identified binding sites. The 3D structure of suramin is shown as sticks in red. Figures were generated by PyMOL. (**a**) Surface hydrophobicity of hRKIP; (**b**) Electrostatic surface of hRKIP. (**c**) Interactions between hRKIP and suramin produced by Ligplot based on the docked structural model of hRKIP-suramin. (**d**) Relative affinities of suramin binding to the mutants relative to hRKIP. The affinity of hRKIP was set to 100.

To further disclose the structural basis of hRKIP binding suramin, we established a structural model of the hRKIP-suramin complex based on the identified binding sites described above. In the docked structural model, suramin roughly adopts a V shape-like conformation to fit into the conserved ligand-binding pocket of hRKIP (Figure 3a). K113 is crucial for hRKIP binding suramin, its amine group of K113 forms a π-cation interaction with the naphthalene ring of suramin, and it also forms two hydrogen bonds with two sulfonic groups of suramin (Figure 3b,c). Moreover, both K113 and W173 form hydrophobic interactions with the naphthalene ring of suramin, and P112 also forms hydrophobic interactions with the phenyl ring adjacent to the urea group of suramin (Figure 3a,c). Note that P112 is located at the conserved ligand-binding pocket together with the residues P74, Y81, S109, and P111. Additionally, the aromatic ring of Y181 forms a hydrogen bond with one amide group of suramin (Figure 3c).

To further verify the substantial interactions of these residues with suramin which were identified from the established structural model, we prepared eight single point-mutated hRKIP proteins, including P74L, Y81A, S109A, P111L, P112L, K113A, W173A, and Y181A. We performed BLI experiments to quantitatively compare the affinities of suramin binding to hRKIP and its mutants (Figure 3d, Table 1). The results showed that the three mutations of K113A, W173A, and Y181A largely decreased the affinities by more than 75%, suggesting that K113, W173 and Y181 play decisive roles in hRKIP binding suramin. Differently, P74L mutant decreased the affinity more than 30%, andY81A decreased the affinities around 20%, implying that P74 and Y81 might also be important for the interaction of hRKIP with suramin. However, S109 had little effect on the binding of suramin to hRKIP for its mutation only decreased the binding affinity around 10%. Interestingly, the P112L mutant showed a slightly increased affinity, potentially owing to the enhanced hydrophobic interaction of L112 with hRKIP. As expected, leucine has a larger hydrophobic sidechain than proline. Unfortunately, the affinity of the P111L mutant was not measured since it was not solubly expressed in *E. coli*.

### 2.4. Suramin Enhances the Phosphorylation of ERK

**Figure 4:**
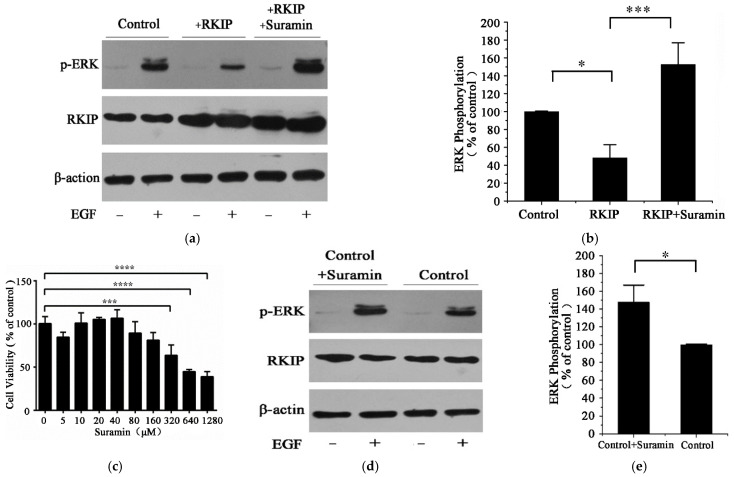
Effects of suramin treatment on hRKIP-mediated Ras/Raf1/MEK/ERK pathway in HEK293 cells (**a**,**b**) Western blot analysis for the effect of suramin treatment on hRKIP-mediated ERK phosphorylation;(**c**) Effect of suramin treatment on viabilities of HEK293T cells transfected with hRKIP. (**d**,**e**) Western blot analysis for the effect of suramin on the ERK phosphorylation of HEK293T cell. * *p* <0.1; *** *p* <0.001; **** *p* <0.0001, *n* = 3 for each group.

We further explored the effects of suramin treatment on hRKIP-mediated Ras/Raf1/MEK/ERK signal pathway by detecting the phosphorylation level of ERK, which could only be activated by MEK [37]. Principally, hRKIP binding with Raf1 inhibits the phosphorylation of Raf1, thereby blocking the following ERK phosphorylation and downregulating the MAPK pathway. As expected, human HEK293T cells with hRKIP transfection showed a 58% decrease in the phosphorylation level of ERK relative to control cells without hRKIP transfection (Figure 4a,b). The transfected cells were then treated with 160 μM suramin. The suramin concentration was experimentally determined as the maximal tolerant concentration for cell viability (Figure 4c), which was consistent with that used usually in clinical patients [12]. We also determined the IC_50_ value of suramin to HEK293T cell was 479 μM by MTS experiments, and found 160 μM suramin could enhanced the phosphorylation level of p-ERK around 48% (Figure 4d,e). While the phosphorylation level of ERK in the hRKIP-transfected cells with suramin treatment was significantly increased by around 3 times compared with that without suramin treatment. The enhanced phosphorylation level suggested that the transfected cells with suramin treatment might greatly reduce the number of hRKIP molecules binding Raf1, thereby relieving the inhibitory role of hRKIP in the Ras/Raf/MEK/ERK pathway (Figure 4a,b). In other words, suramin bound to the conserved ligand-binding pocket of hRKIP, and thus profoundly inhibited the interaction of hRKIP with hRaf1. As a result, the free Raf1 kinase was phosphorylated and the Ras/Raf1/MEK/ERK pathway was promoted with an enhanced phosphorylation level of the following ERK kinase.

Summarily, we have identified hRKIP to be a novel binding target of suramin. Suramin binds to the conserved ligand-binding pocket of hRKIP and prevents hRKIP from binding with hRaf1, thus promoting the MAPK pathway. The clinical treatment with suramin would potentially relieve the inhibitory role of hRKIP in Ras/Raf1/MEK/ERK signaling and promote the MAPK pathway. This work reveals the structural basis of suramin interacting with hRKIP, providing a mechanistic understanding for the clinical side-effects of suramin. Our results may be beneficial to improving suramin and designing suramin derivatives with more isoform-specificity and less side-effects. In future, structural determination of the hRKIP-suramin complex is needed to achieve more in-deep understanding of crucial roles that suramin plays in the RKIP-mediated signal pathway.

## 3. Materials and Methods

### 3.1. Protein Expression and Purification

Both unlabeled and ^15^N-labeled hRKIP proteins were prepared as described previously [35]. Eight hRKIP mutants (P74L, Y81A, S109A, P111L, P112L, K113A, W173A, and Y181A) were generated by site-directed mutagenesis using the vector of pReceiver-B01a-RKIP as the template and confirmed by DNA sequencing. All protein purifications were performed at 4 °C. Protein purities were verified by polyacrylamide gel electrophoresis, and protein concentrations were measured by NanoVue (GE Heathcare Co, Boston, MA, USA).

### 3.2. Biolayer Interferometry (BLI) Assays

The interaction between hRKIP and suramin was detected by using the ForteBio Octet Red system (ForteBio, Inc, San Francisco, CA, USA). The wild-type His-tagged hRKIP protein or its mutant was immobilized onto NTA reactive biosensors, and the captured biosensors were individually dipped into the wells containing various concentrations of suramin (25, 50, 100, 200, and 400 μM). The association at each suramin concentration was detected for 900 s, while the dissociation was monitored for 1800 s. The sensors loading hRKIP without suramin were used as controls to correct baseline drifts. The wells containing various concentrations of suramin without loading protein served as references. The association and dissociation responses were corrected and processed using Octet Data Analysis Software (Version 7.0, ForteBio, Inc, San Francisco, CA, USA). The interferometry data were fitted using the 1:1 Langmuir binding model. 

### 3.3. Nuclear Magnetic Resonance (NMR) Spectroscopy

2D ^1^H-^15^N HSQC spectra were recorded at 25 °C on a Bruker Avance III 600 MHz spectrometer with a TCI cryoprobe. Titration experiments were performed by adding the stock solution of 150 mM suramin to ^15^N-labeled hRKIP sample to obtain 1:1 molar ratio of hRKIP: suramin. NMR spectra were processed and analyzed using the CCPNmr software [38]. NMR assignments of hRKIP were performed previously by our group [35] and deposited into the Biological Magnetic Resonance Bank (BMRB code: 16992, Madison, WI, USA).

### 3.4. Molecular Docking

The GOLD (Genetic Optimization for Ligand Docking) program (CCDC, Cambridge, UK) was applied to establish the structural model of the hRKIP-suramin complex [39]. The three-dimensional (3D) structure of hRKIP in solution was previously determined by our group [23] and deposited into the Protein Data Bank (PDB ID: 2L7W, http://www1.rcsb.org/ (accessed on 25 January 2021)). The binding sites of suramin on hRKIP were defined based on the coordinate centers of amino acids which experienced significant peak broadens in 2D ^1^H-^15^N HSQC spectra. For the molecular docking, 20 genetic algorithm runs were used with the possibility of early termination at each round. The search efficiency of the genetic algorithm was set at the default 100% setting. Qualities of the docked structural models were assessed with the ChemPLP scoring function (CCDC, Cambridge, UK) [40]. The lowest-energy structural model with the highest score was used to mechanistically analyze the interaction between hRKIP and suramin. Structural representations were prepared by the PyMOL program (Schrödinger, LLC, New York, NY, USA).

### 3.5. ERK Phosphorylation Analyses

Human HEK293T cells were used as control group. HEK293T cells were first transfected with the exogenous hRKIP gene (the hRKIP-treated group), then the hRKIP transfected cells were serum starved overnight (10 h) and then incubated with 160 μM suramin for 30 min before EGF stimulation (the hRKIP-suramin-treated group). The transfection process was performed as following: 4 μg of pcDNA3.0-hRKIP was mixed with 75 μL lipotap (Beyotime, China), and the mixture was incubated with HEK293T cell in the dulbecco’s modified eagle medium (DMEM) with 100 units/mL penicillin (Sagon, Shanghai, China), 100 μg/mL streptomycin (Sagon, Shanghai, China) and 10% fetal bovine serum (Hyclone, Logan, UT, USA) for 6 h. Then the lipotap-DNA mixture was removed, and the transfected cell was further cultured in fresh medium. For all the three groups, the cells were cultured in DMEM supplemented with serum and antibodies at 37 °C. At 70% confluency, 10 ng/mL EGF was used to stimulate the Ras/Raf/MEK/ERK signal pathway for 5 min. The reaction was stopped by phosphate-buffered saline. The cells were lysed with a lysis buffer (20 mM Tris-HCl, pH 7.5, 150 mM NaCl, 1 mM Na_2_EDTA, 1 mM EGTA, 1% Triton, 2.5 mM sodium pyrophosphate, 1 mM beta-glycerophosphate, 1 mM Na_3_VO_4_, 1 µg/mL leupeptin and 1 mM PMSF) for 15 min. Thereafter, the supernatant was collected by centrifugation at 11,000 rpm for 15 min and subjected to Western blotting analysis. Western blotting was conducted following a standard procedure as described previously [41]. The corresponding antibodies included Rabbit RKIP antibody (CST, Boston, MA, USA), Phospho-p44/42 MAPK antibody (Beyotime, Shanghai, China) and β-actin antibody (Proteintech, Wuhan, Hubei, China). β-actin was used as a loading control. The digital analysis of immunoreactivity was performed with the ImageJ software.

### 3.6. Statistical Analysis

Cellular assay data are presented as mean ± standard deviation (SD). Three independent experiments were performed in the assay. Statistical significances were determined using Student’s t-test. *p* < 0.05 was considered statistically significant.

## Figures and Tables

**Table 1:** BLI-derived kinetic parameters describing the interactions of suramin with hRKIP and its mutants.

Protein	K_D_ (×10^−5^ M)	k_on_ (M^−1^s^−1^)	k_off_ (×10^−4^ s^−1^)	k_obs_ (×10^−4^ s^−1^)	Full R^2^ (×10^−1^)
RKIP	2.38	8.69	2.07	36.8	9.68
P74L	3.71	7.39	2.75	3.67	9.92
Y81A	2.97	5.31	1.58	2.91	9.94
S109A	2.60	6.93	1.80	29.5	9.90
P112L	2.04	8.14	1.66	3.70	9.57
K113A	8.72	3.38	2.95	3.80	8.15
W173A	7.73	3.12	2.41	5.53	9.76
Y181A	71.1	0.22	1.56	1.77	9.63

## Data Availability

The data presented in this study are available in this article.

## Sample Availability

Samples of the compounds are not available from the authors.
